# Radial Migration Dynamics Is Modulated in a Laminar and Area-Specific Manner During Primate Corticogenesis

**DOI:** 10.3389/fcell.2020.588814

**Published:** 2020-10-16

**Authors:** Veronique Cortay, Delphine Delaunay, Dorothée Patti, Elodie Gautier, Nathalie Doerflinger, Pascale Giroud, Kenneth Knoblauch, Cyril Huissoud, Henry Kennedy, Colette Dehay

**Affiliations:** ^1^University of Lyon, Université Claude Bernard Lyon 1, Inserm, Stem Cell and Brain Research Institute U1208, Bron, France; ^2^Service de Gynécologie-Obstétrique, Hôpital de la Croix-Rousse, Hospices Civils de Lyon, Lyon, France; ^3^Institute of Neuroscience, State Key Laboratory of Neuroscience, Key Laboratory of Primate Neurobiology, Chinese Academy of Sciences, Shanghai, China

**Keywords:** cerebral cortex, macaque, area-specific, supragranular neurons, migration

## Abstract

The orderly radial migration of cortical neurons from their birthplace in the germinal zones to their final destination in the cortical plate is a prerequisite for the functional assembly of microcircuits in the neocortex. Rodent and primate corticogenesis differ both quantitatively and qualitatively, particularly with respect to the generation of neurons of the supragranular layers. Marked area differences in the outer subventricular zone progenitor cell density impact the radial glia scaffold compactness which is likely to induce area differences in radial migration strategy. Here, we describe specific features of radial migration in the non-human primate, including the absence of the premigratory multipolar stage found in rodents. *Ex vivo* approaches in the embryonic macaque monkey visual cortex, show that migrating neurons destined for supragranular and infragranular layers exhibit significant differences in morphology and velocity. Migrating neurons destined for the supragranular layers show a more complex bipolar morphology and higher motility rates than do infragranular neurons. There are area differences in the gross morphology and membrane growth behavior of the tip of the leading process. In the subplate compartment migrating neurons destined for the supragranular layers of presumptive area 17 exhibit radial constrained trajectories and leading processes with filopodia, which contrast with the meandering trajectories and leading processes capped by lamellipodia observed in the migrating neurons destined for presumptive area 18. Together these results present evidence that migrating neurons may exhibit autonomy and in addition show marked area-specific differences. We hypothesize that the low motility and high radial trajectory of area 17 migrating neurons contribute to the unique structural features of this area.

## Introduction

Radial migration of glutamatergic neurons from their birthplace in the germinal zones (GZ) to their final destination in the cortical plate (CP) is a complex process requiring a series of highly coordinated cellular events. Pasko Rakic established the crucial role of radial migration- where pyramidal glutamatergic neurons follow a trajectory that is perpendicular to the ventricular surface, and parallel to radial glial fibers (Rakic, 1972; Misson et al., 1991)-thereby translating the topography of the proliferative fate map in the ventricular zone to the CP (Rakic, 1988; Dehay et al., 1993; Polleux et al., 1997). Birthdating studies showed that layers II–VI of the cerebral cortex are generated in an “inside-out” sequence (Rakic, 1974). Neurons generated early reside in deeper infragranular (IG) layers (layers 5, 6), whereas late born neurons after completing their migration form the superficial, supragranular (SG) layers (layers 2–4).

Several studies have shown that electrical coupling between sister excitatory neurons ensures an important early step in the functional development of the cortex (Yu et al., 2009, 2012; Li Y. et al., 2012). During their radial migration sister excitatory neurons progressively and selectively form gap junctions with each other (He et al., 2015). These observations suggest that the spatial precision of radial migration is a key determinant of highly specific neuronal connectivity as has been shown in the spinal cord (Surmeli et al., 2011). These observations suggest that the area differences in progenitor cell and radial glia scaffold densities could require different migration strategies (Lukaszewicz et al., 2005; Betizeau et al., 2013).

In primates there are two features which could lead to differences in the migrational strategies with respect to those observed in rodents. Firstly, there is a massive increase in the thickness of the cortical subplate (SP), a partially transient compartment of the embryonic neuroepithelium (Kostovic and Rakic, 1990; Smart et al., 2002). The cortical SP increases throughout corticogenesis reaching its maximum extent during the final stages of corticogenesis when the SG layers are being generated. This results in the migratory trajectory of SG layers neurons in primates compared to rodents being multiplied by at least a factor of 10, which could be expected to lead to adaptive mechanisms to ensure accurate and timely arrival of postmitotic neurons to their final location in the brain. Secondly, in the primate, the temporal sequence of rates of neuronal production departs largely from that of the rodents. While in the rodent cortical progenitors show declining proliferative capacities overtime (Smart, 1972; Takahashi et al., 1995a,b), primate cortical neuron production is characterized by a late upsurge of proliferation of the outer subventricular zone (OSVZ) progenitors generating the SG layers (Lukaszewicz et al., 2005; Dehay and Kennedy, 2007; Hansen et al., 2010; Betizeau et al., 2013). This increased SG neuron production is responsible for the selective enlargement of SG layers in the human and non-human primate (Hutsler et al., 2005; Betizeau et al., 2013), which in turn could play a significant role in the increased computational and cognitive abilities in this order (Harris and Mrsic-Flogel, 2013).

Because rates of neuron production and migration have to be coupled to ensure a coherent process, the spatiotemporal variations in neuron production rates combined with changing migratory distances for different populations of neurons might put important constraints on neuron migration. Hence, we hypothesize that differences in the tempo of neuron production will require regulation of radial migration dynamics, that can be optimally explored by comparing neuron migration across layers and areas. Given the marked differences in rates of neurogenesis in visual areas 17 and 18 we have explored the morphodynamic properties of IG and SG migrating neurons traversing the subplate (SP) of these two areas (Lukaszewicz et al., 2005, 2006).

Based on *ex vivo* observations of organotypic slices of embryonic macaque cortex, we provide evidence of primate-specific features of migration. In particular, we show that primate early postmitotic neurons exhibit a bipolar morphology at the pre-migratory stage, in sharp contrast with the multipolar shape described in rodents (Tabata et al., 2009). We describe distinct morphodynamic features of IG and SG neurons during radial migration on organotypic slices of embryonic cortex. Migrating SG neurons exhibit a more complex gross morphology and significantly higher motility rates than do IG neurons in both areas. Remarkably, A17 and A18 SG neurons exhibit different radial migration strategies: whereas A17 neurons migrate according to a radial axis, A18 SG neurons follow curvilinear, ab-radial trajectories. Using an *in vitro* assay, which recapitulates the area-specific differences in migration trajectories, we further characterize the morphology of radial migrating neurons in both areas. Specifically, we identified distinctive features of the growing tip of the leading process in SG migrating neurons from A17 and A18 that we hypothesize could be related to the radial and ab-radial modes of migration. The fact that the area differences in gross morphology along with differences in area migration strategy were preserved *in vitro*, point to cell autonomous properties.

In sum, the comparative analysis of migrating neuronal populations to different layers (IG and SG) and areas (A17 and A18) highlights laminar and area-specific characteristics of radial migratory rates and trajectories in the primate cortex.

## Materials and Methods

### Primates

The cynomolgus monkey (*Macaca fascicularis*) facility in this study and all experimental protocols were approved by the Animal Care and Use Committee CELYNE (C2EA#42). The animals were housed in a controlled environment (temperature: 22 ± 1°C) with 12 h light/12 h dark cycle (lights on at 08:00 a.m.). All animals were given commercial monkey diet twice a day with tap water *ad libitum* and were fed fruits and vegetables once daily. During and after experiments, monkeys have been under careful veterinary oversight to ensure good health. Fetuses from timed-pregnant cynomolgus monkeys (*M. fascicularis*) were delivered by cesarean section according to protocols described in Lukaszewicz et al. (2005). Surgical procedures and animal experimentation were in accordance with European requirements 2010/63/UE. Protocols C2EA42-12-11-0402-003 and APAFIS#3183 have been approved by the Animal Care and Use Committee CELYNE (C2EA #42).

### Plasmids

pCMV-EGFP retrovirus (Betizeau et al., 2013) were produced by M. Afanassieff (SBRI, INSERM U1208 Bron, France) via pTG5349, pTG13077 (Transgene SA, Illkirch-Graffenstaden, France), and phCMV-G [Gift from D. Nègre ENS Lyon (Yee et al., 1994)].

mCherry construct is as follows. NheI-mcherry-XhoI PCR of mCherry cDNA, from pmCherry-N1 plasmid from Clontech (PT3974-5), was cloned into modified pEGFPC1 plasmid from Clontech (ref 6084-1) where EGFP-C1 was previously switched for MCS NheI-SmaI-EcoRV-ClaI-XhoI.

### Organotypic Slice Culture

Lethally anesthetized E63-E65 and E77-E80 fetuses were perfused through the heart with cold supplemented HBSS (Gibco, 14180046) (HBSS with glucose 18%, MgSO4 and CaCl2).

Occipital poles of embryonic hemispheres were isolated and embedded in 3% low-gelling agarose (Sigma, A9045) in supplemented HBSS at 37°C. 300 μm-thick parasagittal slices were cut in 4°C supplemented HBSS using a vibrating blade microtome (Leica VT1000 S). Slices were mounted on Laminin (10 μg/ml)/Poly-L-lysine (100 μg/ml) (Sigma, L2020 and P1399) coated 0.4 μm Millicell Culture Insert (Millipore, PICM0RG50) on a drop of type I collagen (BD Biosciences, 354236). Slices were cultured at 37°C and 7.5% CO_2_, in 6-well plates in 1.2 mL of GMEM/10% FCS: Glasgow minimum essential medium (GMEM, Gibco, 21710-025) supplemented with 1% sodium pyruvate (Gibco, 11360-039), 100 μM beta-mercapto-ethanol (Gibco, 31350-010), 1% non-essential amino acids (Gibco, 11140-035), 2 mM glutamine, 1% penicillin/streptomycin (Gibco, 10378-016), and 10% fetal calf serum (FCS, Pan Biotech, P30-2600). Culture medium was renewed twice a day.

### Retroviral Infection in Embryonic Primate Cortex

Cycling progenitors in the germinal zones were infected with a pCMV-EGFP retrovirus. Floating E63-E65 and E77-E80 cortical slices (300 μm thick) were incubated in GMEM (Gibco, 21710-025) culture medium containing pCMV-EGFP retrovirus (1–5.10^5^ pi/mL), for 2–3 h at 37°C. The slices were then mounted on a Millicell Culture Insert system on a drop of type I collagen (see above for detailed procedure).

### Dissociated Subplate (SP) Cell Culture and Lipofection

The SP was isolated from parasagittal slices via manual microdissection. SP were dissociated with trypsin 1X (Gibco, 1540054) for 3 min at 37°C, manually triturated, washed in GMEM/10% FCS and centrifuged for 5 min at 1000 rpm. Individual cells were plated at 5.10^4^ cells per well on poly-L-Lysine (Sigma, P1399,100 μg/ml)/Laminin (Sigma, L2020, 10 μg/ml) coated lab-tek (Thermo Scientific, 155409) or at 1.10^5^ cells on 14 mm diameter poly-L-Lysine (100 μg/ml) /Laminin (10 μg/ml) coated glass cover slips. Cells were maintained for 1 DIV in GMEM/10% FCS medium, before being transfered into Neurobasal A medium (Gibco, 10888-022) supplemented with B27 (1:50^*e*^, Gibco, 17504-044), N2 (1:100^*e*^, Gibco, 17502-048) and PSG (1X, Gibco, 10378-016) and maintained at 37°C in 7.5% of CO2.

Dissociated neurons were lipofected with the mCherry plasmid using Lipofectamine 2000 (Invitrogen, 11668019) according to the manufacturer procedures.

### Neurosphere Assay

A17 and A18 GZ were isolated from E77 to E80 parasagittal organotypic slices via manual microdissection and cells dissociated using TrypLE Express 1X (Thermo Fisher, 12604013). For each area, 1–2.10^6^ cells were diluted in 4 ml of NS medium, DMEM:F12, (Gibco, 31331-028), N2 supplement, (1:100^*e*^, Gibco, 17502-048), 1% Non-essential amino acids (Gibco, 11140-035), 2 mM glutamine, 1% penicillin/streptomycin (Gibco, 10378-016), 100 μM beta-mercapto-ethanol (Gibco, 31350-010), 20 ng/ml bFGF, (Millipore, GF003AF), 20 ng/ml EGF, (Millipore, 01-107), 1000 U/ml human recombinant LIF, (homemade, Gift from P. Savatier) and grown as neurospheres for 2–10 days in a 50 mm Petri Dish. Neurospheres were then plated on 6 or 24 glass well plates or on 14 diameter glass coverslips coated on Laminin (10 μg/ml)/Poly-L-lysine (100 μg/ml) (Sigma, L2020 and P1399), and allowed to differentiate in Neurobasal A medium supplemented with B27 (1:50^*e*^, Gibco, 17504-044), N2 (1:100^*e*^, Gibco, 17502-048) and 2 mM glutamine, 1% penicillin/streptomycin (Gibco, 10378-016).

For live recording: Two to five days after plating, neurospheres on glass well plates were imaged under the time lapse for 5–7 days. Neurospheres were fixed at the end of recording session with 2% PFA and immunostained for Ki67 (Neomarker, clone sp6, RM9106S1) and NeuN (Millipore, MAB377). For A17 and A18 experiments, NeuN^+^ /Ki67^–^ post-mitotic neurons were tracked.

### Brain and Organotypic Slices Cryosections

For organotypic cortical slices (300 μm thickness), cultured slices were fixed 1h by immersion in cold buffered 2% paraformaldehyde and then cryoprotected in 10 and 20% sucrose. For whole brain cryosections, lethally anesthetized primate fetuses (via intraperitoneal injection of Sodium Pentobarbital 60 mg/kg) were perfused through the heart with buffered 4% Paraformaldehyde (PFA) during 30 min. After sequential cryoprotection in 10% and 20% sucrose (in phosphate buffer), brains were embedded in Tissue-Tek. Immunolabelling against Vimentin was performed on either 80 μm thick organotypic slices (Figure 3I) or on 20 μm thick parasagittal sections performed with a cryostat (Microm, HM550) then mounted on superfrost glass slides (Superfrost Plus, Thermo Scientific) and stored à −20°C (Figure 3J).

### Immunofluorescence, Antibodies and Confocal Imaging

Cryosections were air-dried for 30 min and hydrated in Tris-buffered saline (TBS) for 30 min. Glass slides or coverslips were rinsed three times in TBS Triton (0.5%) and incubated in Normal Goat Serum 10%, (Gibco, 16210-064) diluted in Dako Diluent (Dako, S3022) for 30 min. Primary antibodies were incubated overnight in Dako Diluent at 4°C. Chicken anti-EGFP (Invitrogen, A10262, 1:1000), mouse anti-Vimentin (Sigma, V6630, 1:400), mouse anti-NeuN (Millipore, MAB377, 1:100), Rabbit anti Ki67 (Neomarker, clone sp6, RM9106S1, 1:400). After 3 TBS wash, relevant secondary antibodies were incubated in Dako Diluent (Dako, S3022) 1 h at RT, at the following concentrations: Alexa Fluor 488 goat anti-chicken IgY (Invitrogen, A11039, 1:1000), Alexa Fluor 555 goat anti-mouse IgG (Invitrogen, A21422, 1:800), Alexa Fluor 488 goat anti-mouse IgG (Invitrogen, A11001, 1:1000), Alexa Fluor 555 goat anti-rabbit IgG (Invitrogen, A21428, 1:800). Nuclear staining was performed using DAPI (Invitrogen, D1306, 3 μM in TBS), for 10 min at RT. Mounting was realized in Fluoromount-G Medium (Southern Biotech, 0100-01).

Confocal examination of the fluorescent labeling was carried out on a LEICA DM 6000 CS SP5 equipped with an Argon laser tuned to 488 nm, a HeNe laser 543 nm, a HeNe laser 633 nm, and a diode 405 nm. Acquisition were performed using oil objectives (×40), thanks to the LAS AF software (Leica).

### Two-Photon Time-Lapse Video Recordings on Organotypic Slices

Real time video recordings were performed on an inverted Axio-Observer Z1 (Zeiss) two-photon microscope, equipped with Zeiss optics and a Chameleon system Ultra (I) Titanium Sapphire 80 MHz laser. The recording system is equipped with a Microscope Cage Incubation System (Okolab) maintaining temperature at 37°C and CO_2_ at 7.5%. Millicell inserts were imaged in a 6-well glass bottom plate (Iwaki, #5816-006). The medium was renewed twice a day. Laser was tuned to 910 nm for EGFP imaging (power range 14–20%). Observations were performed using a plan apochromatic dry objective 10×/0.45 with a digital zoom of 1.5. Video-analysis was initiated 72 h after EGFP retroviral infection (E65) and 96 h post infection (E78). Using the Multi Time Series macro of Zeiss Zen software, 4D stacks were acquired over 80 μm thickness (14 optical sections spaced at 6 μm intervals), which allows following of the 4D migration pattern of progenitors and postmitotic neurons.

Recording was performed using a single scanning run at 1024 × 1024 pixels resolution with a scanning speed of 6 μsec/pix. Images were acquired every 1.5 h for up to 15 days.

### Time-Lapse Recording on Dissociated SP Neurons

Video recordings were performed using a Nikon Eclipse Ti S inverted fluorescence microscope with an Andor Clara camera using a Nikon S ELWD Plan-fluor 40×/0.60 objective. The microscope was equipped with a humidified chamber (Nikon, the Box) and recordings were done at 37°C (Nikon, The Cube) under 7.5% CO2 (Nikon, the Brick). Recordings were made on a single focal plane at a rate of 0.1–2 s for each acquisition over 1 min.

### Time-Lapse Recording on Neurospheres

Video recordings were performed using a Nikon Eclipse Ti S inverted fluorescence microscope with an Andor Clara camera using a 10×/0.3 Nikon plan fluor objective or a Plan fluor 20×/0.45. The microscope is equipped with a humidified chamber (Nikon, the Box) and recordings were done at 37°C (Nikon, The Cube) under 7.5% CO2 (Nikon, the Brick). Alternatively, a Leica DMIRBE inverted microscope was used. Recordings were done at 37°C under 7.5% CO2 (PECON). Phase contrast recordings were made every 10 min for up to 7 days. Half of the culture medium was renewed every day.

### Manual Tracking of Radial Migration on Video Recordings in Organotypic Slices and Neurospheres

#### Organotypic Slices

Tracking of EGFP^+^ migrating neuron soma movements was done manually by the experimenter by using the plugin MTrackJ, from GZ to the SP (a java program developed by Erik Meijering at the Biomedical Imaging Group Rotterdam). A neuron is considered to pause if it covers a distance less than 3.5 μm over a 6 h period. The analysis has been performed in lower two thirds of the SP (up to 850 microns). Typical SP neurons trajectories (Figure 3) are reconstructed using raw coordinates (x,y) obtained by MTrackJ tracking and modeled with a common origin (*x* = 0; *y* = 0) using the R Studio^®^ software or the Excel software.

The radiality index was calculated for each displacement (i.e., movement between two recorded positions) within each track. The radial vector is defined as the axis perpendicular to the horizontal upper limit of the OFL. The radial index corresponds to the ratio between the radial distance (rd) and the distance corresponding to the shortest path (sp) measured between the start of migration and the recorded positions. The radiality index is an indicator of neuron dispersion with respect to strict radial migration.

#### Neurospheres

Cell tracking was performed for the whole migration trajectory: from the time the neurons exit the neurosphere until migration cessation, using the plugin MTrackJ. A neuron was considered to pause if it moves less than 3.5 μm over a 2 h period. Morphological analysis was performed for each track and for each position within a single track. Two main migrating neurons morphotypes were distinguished: (i) neurons exhibiting an elongated bipolar shape, classified as “Bipolar” and neurons exhibiting a multibranched morphology with neurites growing at the rear and on the sides of the soma, classified as “multibranched.” Scarce migrating neurons with a split leading process were also observed in both A17 and A18 neurospheres.

Frequencies of each morphotype are monitored by computing the time spent by the neuron in each morphology with respect to the total migration time. Typical neuronal trajectories are reconstructed using raw coordinates (x,y) obtained by MTrackJ tracking and modeled with a common origin (*x* = 0; *y* = 0) using the R Studio^®^ software or the Excel software.

The radial vector used to calculate the radiality index (cf above) is defined by the radial glia processes orientation.

The straightness index is computed over the entire trajectory and quantifies directional persistence. It corresponds to the ratio of the straight distance between the origin and the endpoint of migration (tsd) divided by the total distance covered by the neuron (td).

### Nuclear Orientation, Process Orientation

The nuclear and process orientation analysis was performed on MTrackJ individual trajectories data. The angles of the nucleus and the process orientation with respect to the radial vector were computed for each movement. In slices, the radial vector is defined by the axis perpendicular to the horizontal upper limit of the OFL (i.e., parallel to the ventricular border). In neurospheres, the radial vector is defined by the radial glia processes orientation. Radially orientated nucleus and processes present a displacement angle close to the radial vector (i.e., 90°).

### IG and SG Morphological Analysis in the Germinal Zones and in the OFL

E64–E65 and E77–E78 organotypic cortical slices (300 μm thick) infected with a pCMV-EGFP retrovirus were fixed 3–7 days after infection and immunostained for GFP. Confocal acquisitions of EGFP^+^ cells were performed using a Leica HC PL Apo immersion oil 20×/0.70 objective with a digital zoom of 3. 40 μm stacks were taken 1 μm apart. The number of processes starting from the soma were determined based on EGFP visualization. Cells were classified as: no process (np), radial (1–2 processes) and multipolar (≥3 processes).

### IG and SG Subplate Neurons Morphological Complexity Assessment Using Sholl Analysis

The Sholl analysis was performed using the Sholl Analysis plugin for ImageJ (Anirvan Ghosh Laboratory, UCSD), on either fixed or live imaged neurons expressing mCherry (*in vitro*) or cytoplasmic EGFP (*ex vivo*). The following parameters were used: starting radius: 2 μm; ending radius: 100 μm, radius step size: 2 μm.

### Protrusions Analysis

Protrusions on growth cones and neurites of the leading process were analyzed using time lapse video recording of mCherry (pZou-mCherry) lipofected dissociated neurons. Cellular protrusions were scored manually and classified as filopodia (spike-like long protrusions) or lamellipodia (broader sheet-like protrusions). Results were expressed as a percentage of the total protrusions observed in a given neuron population.

### Statistical Analysis of Nucleus and Processes Orientation

Statistical analyses were performed in the R statistical environment (R Core Team, 2017). Generalized Linear Models (GLM) were fit to each experiment with a Poisson family and the canonical log link function. This is equivalent to fitting a log-linear model with a multinomial distribution (Bishop et al., 1975). For the nucleus and process orientation, the linear predictor corresponded to a 6 × 2 (Angle × Experimental Factor) design in which the Experimental Factor was nested within Angle and the model contained no intercept term. These model designs generated pre-planned contrasts in which the differences of the two levels of the Experimental Factor were estimated and tested for significance for each orientation or angle. The possibility of overdispersion was excluded by examining the ratio of the residual deviance to the residual degrees of freedom and by comparing the fits with a negative binomial model, which includes an additional parameter to account for overdispersion. These measures along with examination of model diagnostic plots of the residuals did not reveal any systematic evidence for overdispersion. Statistical significance from the GLM analyses (referred to as GLM test) was evaluated by comparing nested models with and without an interaction of Orientation and the Experimental Factor by likelihood ratio tests and by the Wald statistics for the individual coefficients of the models. *p* < 0.05 was considered statistically significant. Sample number (n) corresponds to the total number of independent biological samples for all the experiments. Data are presented as the mean ± 95% confidence interval (CI).

### Sholl Models and Statistical Analysis

The data for IG and SG neurons and for both conditions (*ex vivo* and *in vitro*) were modeled with the *glm.nb* function from the MASS package (Venables and Ripley, 2002) in the R programming environment (R Core Team, 2017). This fits a GLM with a negative binomial family and a log link function by maximum likelihood. The log volume of the sampling region was used as an offset variable to model estimated densities of the intersections. A segmented linear predictor was used to model the log of the density as a function of distance (Muggeo, 2008). The curves were obtained from the back-transformation of the estimated densities by multiplying them by the volume of the sampling region. The error bars are standard errors of the mean. The correspondence between the *ex-vivo* and the *in vitro* model was evaluated by comparing the log ratio of SG to IG densities among the two conditions.

## Results

**FIGURE 1:**
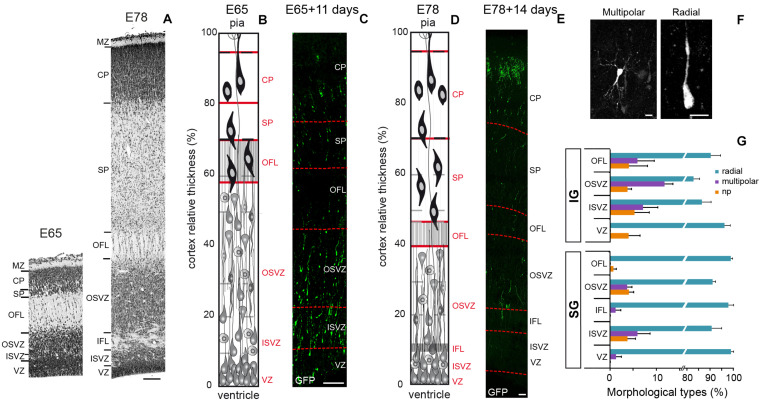
**(A)** Transects of Area 17 at E65 (left) and E78 (Right). **(B)** Area 17 cellular organization at E65. **(C)** Transect of a A17 parasagittal organotypic slice 11 days after EGFP retroviral infection of cycling precursors at E65. A fraction of EGFP^+^ neurons have migrated and reached the CP. **(D)** Area 17 cellular organization at E78. **(E)** Transect of a A17 parasagittal organotypic slice 14 days after retroviral infection at E78. EGFP^+^ neurons have reached the CP. **(F)** Microphotographs of EGFP^+^ neurons at the OFL/SP border showing multipolar (left panel) and radial morphology (right panel). **(G)** Frequency of the three morphological classes of EGFP^+^ early postmitotic migrating neurons in the occipital cortex subsequently to retroviral infection at E65 and E78, respectively: no process (np), radial (1–2 processes), and multipolar (≥3 processes). VZ, ventricular zone; ISVZ, inner subventricular zone; IFL, inner fiber layer; OSVZ, outer subventricular zone; OFL, outer fiber layer; SP, subplate; CP, cortical plate; MZ, marginal zone. Scale bars: **A,C,E:** 100 μm; **F:** 10 μm.

The present study focuses on two developmental stages: E65 and E78 (Figure 1A) that correspond, respectively to the generation of the bulk of IG *(E55–E71)* and SG layer neurons *(E72–E90)* in the occipital cortex of the macaque monkey (Rakic, 1972; Dehay et al., 1993; Lukaszewicz et al., 2005; Betizeau et al., 2013). Both stages are prior to initiation of gyrification of the occipital lobe (Smart et al., 2002). Embryonic organotypic cortical slices provide an unrivaled *ex vivo* non-human primate model of early corticogenesis, where morphology, proliferation, differentiation and migration can be explored in an intact cytoarchitecture over a one to 2 week period (Betizeau et al., 2013; Figures 1B–E and Supplementary Figure S1). IG and SG neurons were labeled via EGFP retroviral infection of cycling progenitors [as in Betizeau et al. (2013)] on organotypic slices from the most posterior pole of the occipital lobe cut in the parasagittal plane, encompassing the primary visual area (area 17 -A17) and its neighbor, area18 (A18) at E65 and E78 (Supplementary Figure S2).

### Early Radially Migrating Neurons Do Not Go Through a Multipolar Stage in the Primate Cortex

We first analyzed the behavior and morphology of IG and SG postmitotic neurons during the pre-migratory stage in the GZ of A17 and A18. Morphological attributes of early postmitotic neurons have been extensively described in the mouse (Nadarajah and Parnavelas, 2002; Tabata et al., 2012). Immediately after cell-cycle exit, mouse postmitotic neurons enter a static stage when they pause for 48 h and assume a multipolar morphology in the SVZ, prior to resuming a bipolar morphology and reinitiating radial migration. Real time observations in the NHP embryonic slices showed that early postmitotic EGFP+ neurons pause for 24–40 h before reinitiating radial migration within the GZ. Upon exit from the OSVZ, newborn radially migrating neurons are characterized by a bipolar, elongated morphology of the nucleus and the soma. The vast majority (>85%) of pre-migratory neurons exhibit a bipolar morphology, and multipolar neurons account only for 12% of IG and 4% of SG premigratory pausing neurons (Figures 1F,G), indicating that the multipolar shape is rare in the germinal zones. This contrasts with the high (88%) proportion of multipolar premigratory postmitotic neurons observed in the SVZ/IZ compartment at mid-corticogenesis in the mouse (Tabata and Nakajima, 2003).

### IG and SG Radial Migrating Neurons Show Distinct Morphodynamic Features

**FIGURE 2:**
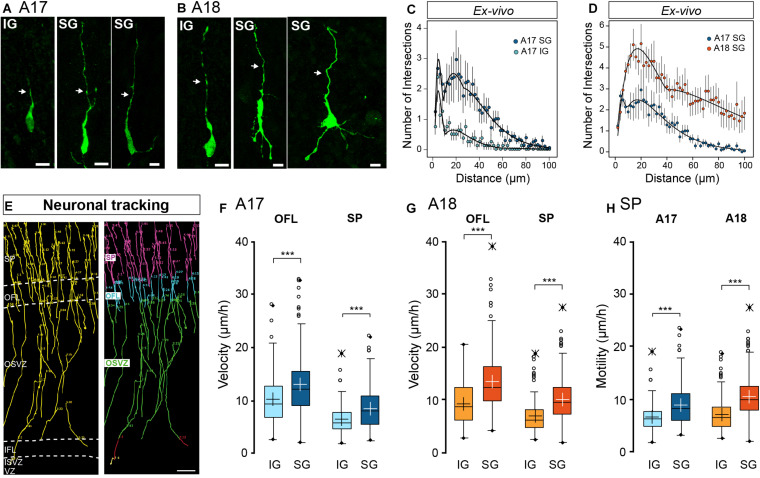
**(A,B)** Characteristic neuronal morphology of A17 **(A)** and A18 **(B)** IG and SG in the SP revealed by GFP immunolabelling. Leading process is highlighted by a white arrow. **(C)** Sholl analysis comparison between A17 IG (light blue) and A17 SG (dark blue) **(D)** Sholl analysis comparison between A17 SG neurons (blue dots) and A18 SG neurons (orange dots) *ex vivo* using the Sholl analysis. **(E)** Typical trajectories of migrating SG neurons reconstructed via manual tracking from A17. 2-photon TLV observations of organotypic slices over a 10 days period (see Supplementary Movie S1), right panel: color-coded tracks with respect to cortical compartments Green: OSVZ, Blue: OFL, Pink: SP. **(F)** IG and SG migration velocity in the OFL and SP of A17. **(G)** IG and SG migration velocity in the OFL and SP of A18, **(H)** Motility index in A17 and A18 SP. Black and white crosses indicate the mean. Average values ± sem. Statistical analysis: Two-tailed and unequal variance Student test, *p* values <0.005***. Scale bars: **A,B** = 10 μm, **E** = 100 μm.

In a first instance, we analyzed the gross morphology of EGFP+ radially migrating neurons in the SP at E65 and E78 in A17 and A18 organotypic slices (Figure 2), 4–6 days following retroviral infection. At E65, IG migrating neurons in the SP present a bipolar morphology in both areas (Figures 2A,B). By contrast SG migrating neuron morphology is more complex, as quantified by the Sholl analysis (Figure 2C), exhibiting a bipolar, elongated soma with a single leading process extending in the direction of migration and a variable number of trailing neurites (Figures 2A,B). In A17, SG migrating neurons possess a single trailing neurite opposing the leading process. By contrast in A18 migrating neurons exhibit a multibranched morphology, with several trailing neurites, resulting in a higher complexity as quantified by a Sholl analysis (Figure 2D). No difference in morphological complexity between A17 and A18 IG migrating neurons has been detected (data not shown).

Using 2-photon real time imaging, we monitored the migratory behavior of EGFP+ newborn IG and SG neurons on parasagittal organotypic slices. Importantly, the parasagittal plane of section of the occipital lobe cuts parallel to the radial glia scaffold. Cell movements were recorded every 1.5 h (Supplementary Movie S1) and migration trajectories tracked manually from the GZ to the SP (Figure 2E and Supplementary Movie S1). At both stages, migrating neurons display a saltatory locomotion with pauses following a period of displacement (Supplementary Movie S2) as reported in mouse (Nadarajah and Parnavelas, 2002; Nadarajah et al., 2003; Kriegstein and Noctor, 2004). We focused on the early stages of radial migration and conducted our analysis in the OFL and the lower two thirds of the SP. Both IG and SG neurons follow a radial trajectory in the upper part of the OSVZ as well as in the OFL and the SP (Figure 2E).

Migration kinetics were characterized by measuring (i) migration velocity, defined in terms of units of distance with respect to time; and (ii) motility index defined by units of distance with respect to time minus cumulative pause duration. A neuron was considered to pause when its movement amplitude is less than 3.5 μm in any 6h period. In both areas, SG neurons show significantly increased migratory kinetics compared to IG neurons in both the OFL and the SP (Figures 2F,G). The higher migration velocity of SG compared to IG neurons (Figures 2F,G) is not due to pausing in either the OFL or SP as indicated by increased values of motility (Figure 2H), reflecting intrinsic differences in motility rates of these two populations. We did not observe differences in IG neurons velocity and motility rates between A17 and A18. By contrast SG neurons show significantly higher velocity and motility rates in A18 compared to A17.

These results point to laminar differences in morphodynamic properties of cortical migrating neurons that are conserved across A17 and A18.

### SG Neurons Show Area-Specific Differences in Radial Migration Mode

**FIGURE 3:**
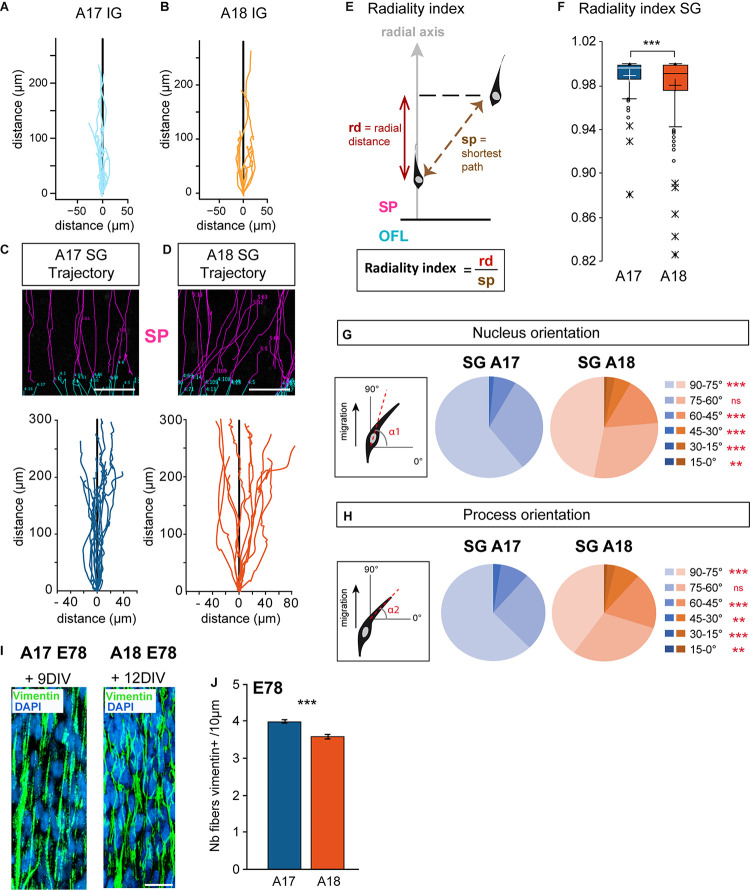
**(A,B)** Trajectories of migrating IG neurons observed in Area 17 and A18 SP using 2-photon TLV on organotypic slices. **(C,D)** Typical radial Area 17 and ab-radial Area18 SG trajectories observed in the SP up to 12 days after retroviral infection. Examples of original SP trackings are presented above the graphs. **(E)** Radiality index. The radiality index corresponds to the ratio between the radial distance (rd) and the distance corresponding to the shortest path (sp) measured between two positions. **(F)** A17 and A18 SG neurons radiality index. Black and white crosses indicate the mean. **(G,H)** Nucleus and leading process changes in orientation during A17 and A18 SG trajectories. Nucleus **(G)** and leading process **(H)** orientations are defined with respect to the surface of the OFL (= 0°) and measured for each recorded position. **(I)** Vimentin expression on A17 and A18 in the lower SP during SG migration, as shown after immunofluorescent labeling on 80 microns thick section from E78 organotypic slices at, respectively, 9–12 days after infection. Nuclei are stained by DAPI. Note the well-preserved radial scaffold. **(J)** Graph showing the density of Vimentin+ fibers in the SP of area 17 and Area 18 per surface area quantified on 20 micron thick sections from E78 cortex. Average values ± sem. Statistical analysis: **(F,J)** Two-tailed and unequal variance Student test, *p* values <0.005***. **(G,H)** GLM test, *p* values <0.01**, <0.001***. SP: subplate. Scale bars: **C,D** = 100 μm, **I** = 20 μm.

We next focused on the analysis of trajectories of individual IG and SG migrating neurons in the SP. Global trajectories were observed to be strictly radial in the IG population in both areas (Figures 3A,B). SG neurons show distinctive behaviors in the two areas. SG migrating neurons in A17 follow relatively linear, radially constrained trajectories. By contrast SG migrating neurons in A18 exhibit meandering, ab-radial trajectories (Figures 3C,D). In order to quantify these differences in SG neurons, we have computed the radiality index (Figure 3E), defined as the ratio between the radial distance with respect to the shortest path between two time points. Deviation values from 1 of the radiality index indicate deviation with respect to the radial axis (perpendicular to the ventricular border). SG migrating neurons in A18 have significantly lower radiality index values compared to SG migrating neurons in A17, indicating that A18 SG neurons significantly deviate from radial routes (Figure 3F).

Migrating neurons in the SP are highly polarized in the direction of migration and exhibit a single leading process exhibiting forward motion. To investigate if the radial and ab-radial trajectories in the SP are associated with differences in cell body morphology and leading process dynamic, we monitored the cell body and the leading process orientations with respect to the radial axis. For each recorded movement in the SP, the cell bodies and leading process of SG migrating neurons in A17 show a high proportion of radial orientations (Figures 3G,H). By contrast, SG migrating neurons in A18 exhibited a significantly lower proportion of radial orientations for both the soma and the leading process (Figures 3G,H).

Our observations were made at stages E65 and E78, which is prior to gyrification, in linear portions of A17 and A18 of the developing visual cortex (Supplementary Figure S2). The radial glia scaffold plays a key role in guiding migrating neurons (Rakic, 1972; Elias et al., 2007; Evsyukova et al., 2013). The tortuous trajectories of migrating A18 neurons at E78 in the SP do not result from an alteration or a disruption of the radial glia scaffold which is preserved over time in organotypic slices (Figure 3I). To further explore whether these area differences in radial and ab-radial trajectories could be due to differences in the radial scaffold density, we quantified the numbers of radial glia processes per surface area in the SP of A17 and A18 (Figure 3J). This shows a higher density of radial glia processes in A17 than in A18, in agreement with the higher numbers of bRGS in A17 OSVZ than in A18 (Lukaszewicz et al., 2005; Betizeau et al., 2013).

### The Leading Process of SG Migrating Neurons Exhibit Area-Specific Features

**FIGURE 4:**
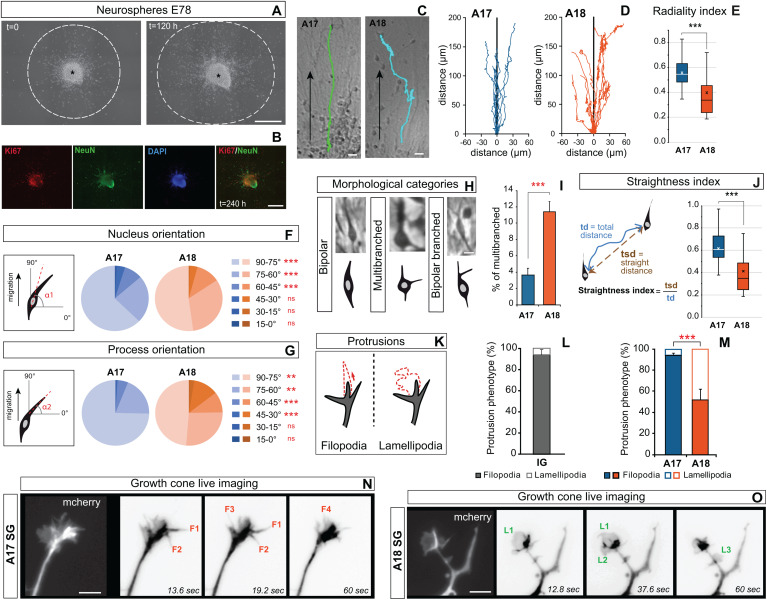
**(A)** Photographs of A18 SG derived neurospheres 4 days after plating, (*t* = 0) (left panel), and 10 days after plating (*t* = 120 h) (right panel) showing neuronal migration extent (dotted line) out of the neurosphere. **(B)** At the end of the live recording, neurons are identified using NeuN and Ki67 immunostaining. NeuN^+^/Ki67^–^ cells, are considered post-mitotic neurons. **(C)** Typical trajectory of A17 and A18 SG neurons migrating out of the neurosphere. **(D)** Typical trajectories of migrating SG neurons reconstructed from time lapse observations. **(E)** Area 17 and Area18 SG neuron trajectories radiality index. **(F,G)** Nucleus **(F)** and process **(G)** change in orientation during A17 and A18 SG neuronal migration out of neurospheres. **(H)** Morphology of SG neurons migrating out of the A17 and A18 neurospheres along their trajectory. Three main categories were identified: bipolar, with two processes located at the opposite poles of the soma; multibranched: with multiple processes growing out of the cell body; bipolar branched: with a branched leading process. **(I)** Percentage of time spent as multibranched for A17 and A18 SG neurons during their migrating trajectory **(J)** The straightness index is computed over the entire trajectory and corresponds to the ratio of the straight distance between the origin and the endpoint of migration (tsd) divided by the total distance covered by the neuron (td). **(J)** Area 17 and Area18 SG neuron trajectories straightness index. Black and white crosses indicate the mean. **(K)** Two types of growing membrane protrusions are observed on the growth cone and/or along the leading process of migrating SP neurons: filopodia (spike-like long protrusions, Left) or lamellipodia (broader sheet-like protrusions, Right). **(L)** Protrusions phenotype frequencies in occipital IG neurons. **(M)** Frequency of filopodia and lamellipodia observed on A17 and A18 SG neurons. **(N)** High magnification protrusions observed on a typical growth cone of an A17 SG SP neuron leading process labeled with mCherry. Imaging took place over 1 min. Four growing protrusions, classified as filopodia **(F1 to F4)**, are highlighted. **(O)** High magnification of a typical growth cone of A18 SG SP neurons labeled with mCherry Three growing protrusions, classified as Lamellipodia **(L1 to L3)** are highlighted. **(E,I,J,M)** Two-tailed and unequal variance Student test, *p* values < 0.005^*⁣**^. **(F,G)** GLM test, *p* values < 0.01^∗∗^, < 0.001^*⁣**^. Scale bars: **A,B:** 500 μm, **C:** 20 μm, **H** = 10 μm, **N,O** = 5 μm.

The differences in the radial glial density in A17 and A18 could mean that the observed area differences in morphology and migration radiality reflect environmental factors. We designed *in vitro* assays in order to achieve high resolution analysis of neuron morphology and membrane behavior at the growing tip of the leading process of migrating neurons derived from cortical progenitors. First, we prepared neurospheres derived from A17 and A18 OSVZ primary GZ progenitors at E78. Four days following plating on a polylysine/laminin coated glass substrate, radial processes were observed to grow evenly out of the neurosphere and postmitotic neurons (Ki67^–^/ NeuN^+^) to migrate out of the sphere (Figures 4A,B). The sparse growth of radial processes emanating from the neurospheres only partially recapitulates cues encountered by the *in vivo* migrating neurons in the SP. Reconstruction of the trajectories of postmitotic neurons migrating out of the neurospheres showed significantly straighter radial paths in migrating neurons derived from A17, compared to tortuous, ab-radial routes in migrating neurons derived from A18 (Figures 4C,D). In addition to the radiality index (Figure 4E), we measured the straightness index, which allows to estimate the tortuosity of migration trajectories (Figure 4J). Both indices are significantly higher in A17 than in A18 neurospheres (Figures 4E,J). In A18, we observed a distinctive pattern of motility where migrating neurons make frequent changes in directionality. Similarly to what we observed during radial migration on organotypic slices, the soma orientation show greater deviation with respect to the radial axis in migrating neurons derived from A18 compared to those derived from A17, a feature that is accentuated in the leading process (Figures 4F,G), on par with what we observed in the organotypic slices (Figures 3G,H). The neurosphere migration assay recapitulates the area-specific distinctive features of SG migrating neurons, in absence of the dense radial scaffold observed *in vivo* and *ex vivo* in the SP, suggesting that these differences are in part expression of cell autonomy.

Having shown that area specificity in migration trajectories are maintained *in vitro*, we checked that differences in gross morphology were also maintained in dissociated neurons, as measured by a Sholl analysis (Supplementary Figure S3). We then took advantage of the higher spatiotemporal resolution provided by *in vitro* dissociated cells, to implement an in-depth analysis of morphology dynamics and leading process behavior. High frequency TLV analysis revealed dynamic changes in the morphology of migrating neurons. We observed two main migrating neurons morphotypes: neurons exhibiting an elongated bipolar shape, classified as “bipolar” and neurons exhibiting a multibranched morphology with trailing and lateral neurites, classified as “multibranched” (Figure 4H). During their migration, migrating neurons derived from A18 show a higher frequency of multibranched morphology than do migrating SG neurons derived from A17, meaning that compared to A17, A18 derived neurons spend a larger fraction of their trajectory in the multibranched state (Figures 4H,I). In addition to these two main morphotypes, we observed scarce migrating neurons with a split leading process emanating from both A17 and A18 neurospheres (Figure 4H), a morphology also seldomly encountered *ex vivo* on the organotypic slices.

Subsequently, we proceeded to characterize the membrane growth behavior of the leading process on dissociated SP migrating neurons. *In vitro*, the leading process of dissociated migrating neurons was identified as the thickest primary process. Membrane protrusions were observed at the growing tip and along the leading process. These protrusions were classified as filopodia (long spike-like protrusions) or lamellipodia (broad sheet-like protrusions) (Figure 4K). Migrating IG neurons in both areas show a predominant filopodia phenotype (Figure 4L). In A17 SG migrating neurons, the vast majority (>95%) of protrusions of the leading process were filopodia (Figures 4M,N). By contrast, A18 SG migrating neurons exhibit up to 50% of lamellipodia which showed curvilinear extension and retraction movements (Figures 4M,O). These area differences observed *in vitro* point to cell autonomous characteristics in membrane behavior at the growing tip of the leading process. In sum, the filopodia membrane growth of the leading process is associated with radial trajectories (IG migrating neurons in A17/A18 and SG migrating neurones in A17), while the lamellipodia protrusion behavior in A18 SG migrating neurons is associated with ab-radial migration trajectory.

## Discussion

### Multipolar Stage Is Dispensable for Primate Cortex Radial Migration

Numerous observations in the rodent cortex (Tabata and Nakajima, 2003; Kriegstein and Noctor, 2004; Noctor et al., 2004; Ohtaka-Maruyama et al., 2018) have reported that before initiating their radial journey toward the CP, around 80% of migrating neurons undergo a transient multipolar phase in the upper SVZ/lower intermediate zone. The present observations in the NHP cortex reveal that multipolar postmitotic neurons represent only a minute fraction of the migrating neurons (4% for SG neurons) and that an overwhelming majority of young migrating neurons exhibit a bipolar morphology. Compared to rodent, the pause duration in macaque between cell-cycle exit and initiation of radial migration corresponds to a considerably reduced window of time. Of note, 90% of the VZ and OSVZ cycling progenitors in the macaque correspond to four morphotypes showing a polarized morphology, which have significantly higher neurogenic potential than the 10% of unpolarized progenitors (Betizeau et al., 2013). This suggests that inheritance of this bipolar morphology establishes the optimal conditions for effective initial steps of radial migration in primate, by contrast with the rodent situation where a large majority of late-born pyramidal precursors are generated from unpolarized intermediate Tbr2 expressing progenitors with multipolar morphologies and highly dynamic processes (Kowalczyk et al., 2009; Kriegstein and Alvarez-Buylla, 2009; Nelson et al., 2013).

### Species-Specific Temporal Regulation of Migration and Proliferation Rates

Despite drastic interspecies variation in the distance to be covered from the GZ to the CP, radial migration rates of cortical neurons appear to be conserved between species. Using real-time imaging on organotypic cortical slices, we observed average radial migration velocities for IG and SG neurons in primate Area 17 and 18 ranging from 6 to 12 μm/h -depending on the developmental stage, compartment and area. These values are in the range reported for bipolar neuron radial migration on organotypic slices of the gyrencephalic cortex of the ferret (Gertz and Kriegstein, 2015) and of the mouse cortex during mid-neurogenesis (Britto et al., 2011; Adnani et al., 2015). Note that the relative invariance of migration speeds between species stands in sharp contrast with the significant interspecies differences in cell-cycle duration (Tc is five times longer in monkey than in mouse-) (Kornack and Rakic, 1998; Lukaszewicz et al., 2005; Betizeau et al., 2013). This suggests that migration speed regulatory mechanisms might be less of an evolutionary target than cell-cycle control mechanisms. The lengthening in migration phase duration between mouse, ferret and primate (Jackson et al., 1989; Noctor et al., 2004; Borrell et al., 2006; Lukaszewicz et al., 2006) is on par with the enlargement of the cortex in these species corresponds to a major evolutionary adaptation offsetting similar rates of migration. This evolutionary constraint in migration rates is likely to result from an exquisitely tuned balance between intrinsic properties and extrinsic factors. Indeed, during their migration journey, IG and SG neurons have to cross different species-specific embryonic compartments, each characterized by distinctive ECM components (Fietz et al., 2010; Ayoub et al., 2011; Zeng et al., 2012).

### Coordinated Regulation of Migration and Proliferation Rates

Given the exquisite correlation between the temporal sequence of cortical neuron birthdates and their laminar distribution in the CP, migration speed might need to be tightly adjusted to rates of neuron production in order to prevent crowding of newborn neurons and to achieve correct neocortical layering. Detailed lineage analysis showed that IG and SG progenitors differ with respect to their proliferative behavior (Betizeau et al., 2013). Specifically SG progenitors have increased proliferative capacities that allow the enlargement of SG layers that characterizes cortical areas in primates (Dehay et al., 2015). Here we find that radial migration velocity of SG neurons is significantly higher than that of IG neuron, on par with the higher production rates of SG neurons. This suggests coordination between proliferation and migration rates. Coordination mechanisms have been described in molecular investigations of neurogenesis and radial migration (Heng et al., 2010; Pacary et al., 2011). In addition, recent data suggest that radially propagative Ca++ activity in radial glial fibers could mediate such a coordination (Rash et al., 2016).

Major rodent-primate differences in corticogenesis may impact radial migration. Throughout rodent corticogenesis, the radial migration scaffold is provided by the basal processes of the apical progenitors (APs) of the VZ that extend both an apical and a basal process anchored to the ventricle and the basal membrane, respectively. During primate corticogenesis, the predominant progenitor pool is the bRGs of the OSVZ (Smart et al., 2002; Lukaszewicz et al., 2005; Lui et al., 2011; Florio and Huttner, 2014). While the vast majority of bRGS extend a long basal process directed to the CP, only a fraction (40%) exhibit an apical process, which is not anchored in the ventricular border (Betizeau et al., 2013). Therefore, one might posit that the scaffolding cues for radial migrating neurons differ between primates and rodents (Nowakowski et al., 2016). The sharp arealization that characterizes the primate cortex is supported by differences in the density of the OSVZ progenitor pool and proliferative programs (Dehay et al., 1993; Smart et al., 2002; Lukaszewicz et al., 2005). The area differences in density of bRGs observed between A17 and A18 (Lukaszewicz et al., 2005), translate into different SP microenvironments for migrating neurons, possibly requiring area-specific regulatory mechanisms.

### Area-Specific Features of Radial Migration

In our experimental design, care has been taken to monitor radial migration in rectilinear regions of cortex in both A17 and A18, prior to folding and away from presumptive sulci and gyri. Hence the ab-radial meandering migration that we observe in A18 SG is not related to a fanning array of the radial glial fibers scaffold associated with gyrification as has been reported in ferret where migration has been studied in presumptive gyri (Borrell et al., 2006; Reillo et al., 2011) or in the GZ during folding (Gertz and Kriegstein, 2015). This is further supported by the observations within the neurosphere migration assay that recapitulates the radial and ab-radial trajectories.

Membrane lamellipodia have been hypothesized to serve as a sensor of the local microenvironment permitting cells to optimize their functional adhesion (Myat et al., 1997; Skalski et al., 2010). The higher occurrence of lamellipodia observed in A18 SG migrating neurons could confer them the ability to probe the environment more efficiently than A17 SG migrating neurons, thereby accommodating the sparser scaffold of radial glial fibers in A18. Compared to A18, the A17 OSVZ expresses higher levels of the primate-specific microRNA miR550-3P at E78 (Arcila et al., 2014). miR550-3p targets srGAP2 (Dennis et al., 2012) which, when overexpressed in mouse cortical progenitors, induces filopodia and highly dynamic membranes with large transient protrusions (Guerrier et al., 2009). Assuming that miR550-3p negatively regulates the expression of srGAP2 leads to the prediction that A17 SG migrating neurons should exhibit less protrusions and lamellipodia than do A18 migrating SG neurons.

We observed that area differences in radial rates of migration as well as the differences in morphology are preserved in dissociated cultures, pointing to intrinsically determined properties of radially migrating SG neurons. How the cell-intrinsic control of the cytoskeleton interfaces with extracellular signal-regulated pathways that control the migration of neurons remains elusive (Pacary et al., 2011). The morphology of the leading process varies in different migrating neuronal types, which is considered to reflect an adaptation to the local migratory requirements (Marin et al., 2010; Valiente and Marin, 2010). While the majority of radially migrating pyramidal neurons exhibit a simple single leading process (Martínez-Martínez et al., 2019), fast tangentially migrating neurons display a more complex leading process with branched morphologies (Bellion et al., 2005). One cannot exclude the possibility that the differences in the morphodynamics of radial migration is partly correlated to changes in geometry and/or adhesive properties of the extracellular environment in the SP. This would reflect an adaptability of migratory strategies in order to maintain high motility as has been reported in other cell models (Tozluoglu et al., 2013, 2015).

Our data point to area-specific features in the trade-off between speed and distance to optimize radial migration efficiency. The tortuous trajectories in A18 SG neurons, indicative of less directional persistence, result in increased path length. This is reminiscent of the migrating behavior of early postmitotic neurons in the GZ in reelin mutants. Compared to control neurons showing a strict radial path, the loss of reelin results in an increased speed and deviation from the rectilinear radial path at the earliest stages of their trajectories (Britto et al., 2011) which is thought to be related to the greater extent of cell dispersion observed in the reeler cortex (Tissir et al., 2002).

### Speculation on the Functional Consequences of Area-Specific Distinct Migratory Strategies

In the present study, our *in vitro* findings indicate that the area differences in migratory characteristics between A17 and A18 postmitotic neurons rely at least in part on cell autonomous mechanisms. This is reminiscent of the area differences in A17 and A18 OSVZ progenitor cell cycle durations that are maintained *in vitro* (Lukaszewicz et al., 2005). Together, these cell autonomous properties suggest that there might be a tight coupling between the proliferative behavior of progenitors and their post mitotic migratory behavior.

In addition to constraining the organization of the ontogenic cortical columns (Rakic, 1988; Jones and Rakic, 2010), the radiality of migration has been shown to influence cortical circuitry assembly in the neocortex. Clonally related neurons have been shown to be preferentially inter-connected and to share functional properties (Yu et al., 2009; Li H. et al., 2012; Ohtsuki et al., 2012; Gao et al., 2014). Induced lateral dispersion of migratory sister excitatory neurons disrupts preferential electrical coupling in the early developing mouse cortex (Yu et al., 2012; He et al., 2015), a crucial early step to ensure its proper functional development (Yuste and Katz, 1991; Elias et al., 2007). The relatively high radial organization of A17 cortical neurons (Rockland and Ichinohe, 2004), which is likely to result from the migration trajectories in the early stages of migration, could therefore have functional consequences on A17 connectivity. In many ways area 17 shows unique properties in terms of adult structure and function that could perhaps require strict radial migration and one could speculate that the slower velocities observed in migrating A17 neurons is the price that has to be paid to achieve maximum radiality. It remains to be determined whether the observed area differences in radial migration modes parallel differences in the local circuitry of primary versus associative areas (Douglas and Martin, 2007). Neuron densities vary across cortical areas (Collins et al., 2010) and SG neurons densities are particularly high in A17 (Rockel et al., 1980). The radial characteristics of migration observed in A17 SG migrating neurons could be linked to the high adult SG neuron density compared to other cortical areas. The spatial modulation of migration so as to accommodate area variation in SG neuron density could be an important adaptive feature given that high densities of SG neurons would favor the sparse coding strategy characteristic of these neurons that is thought to allow enhanced computational capacities (Harris and Mrsic-Flogel, 2013).

## Data Availability Statement

The raw data supporting the conclusions of this article will be made available by the authors, without undue reservation.

## Ethics Statement

The animal study was reviewed and approved by the Comité d’Ethique Lyonnais pour les Neurosciences – CELYNE (C2EA #42).

## Author Contributions

DD, HK, and CD: conceptualization and writing – original draft. VC, DP, and ND: methodology. VC and PG: validation and data curation. KK and VC: formal analysis. VC, DD, DP, EG, and CH: investigation. CH and ND: resources. CD and HK: writing – review and editing. DP, PG, and VC: visualization. CD: supervision, project administration, and funding acquisition. All authors contributed to the article and approved the submitted version.

## Conflict of Interest

The authors declare that the research was conducted in the absence of any commercial or financial relationships that could be construed as a potential conflict of interest.
