# Supplementary material for: Radial Migration Dynamics Is Modulated in a Laminar and Area-Specific Manner During Primate Corticogenesis
Source: Front Cell Dev Biol. 2020 Oct 16;8:588814. doi: 10.3389/fcell.2020.588814 (PMC7596244; doi:10.3389/fcell.2020.588814)
Supplement: Supplementary file 3 [file Presentation_1.pdf]

Supplemental information

Radial migration dynamics is modulated in a laminar and area-specific manner during primate corticogenesis.

Veronique Cortay[1,$], Delphine Delaunay[1,$], Dorothée Patti[1], Elodie Gautier[1], Nathalie Doerflinger[1], Pascale Giroud[1], Kenneth Knoblauch[1], Cyril Huissoud[1,2], Henry Kennedy[1,3] and Colette Dehay[1,*]

1) Supplemental figures

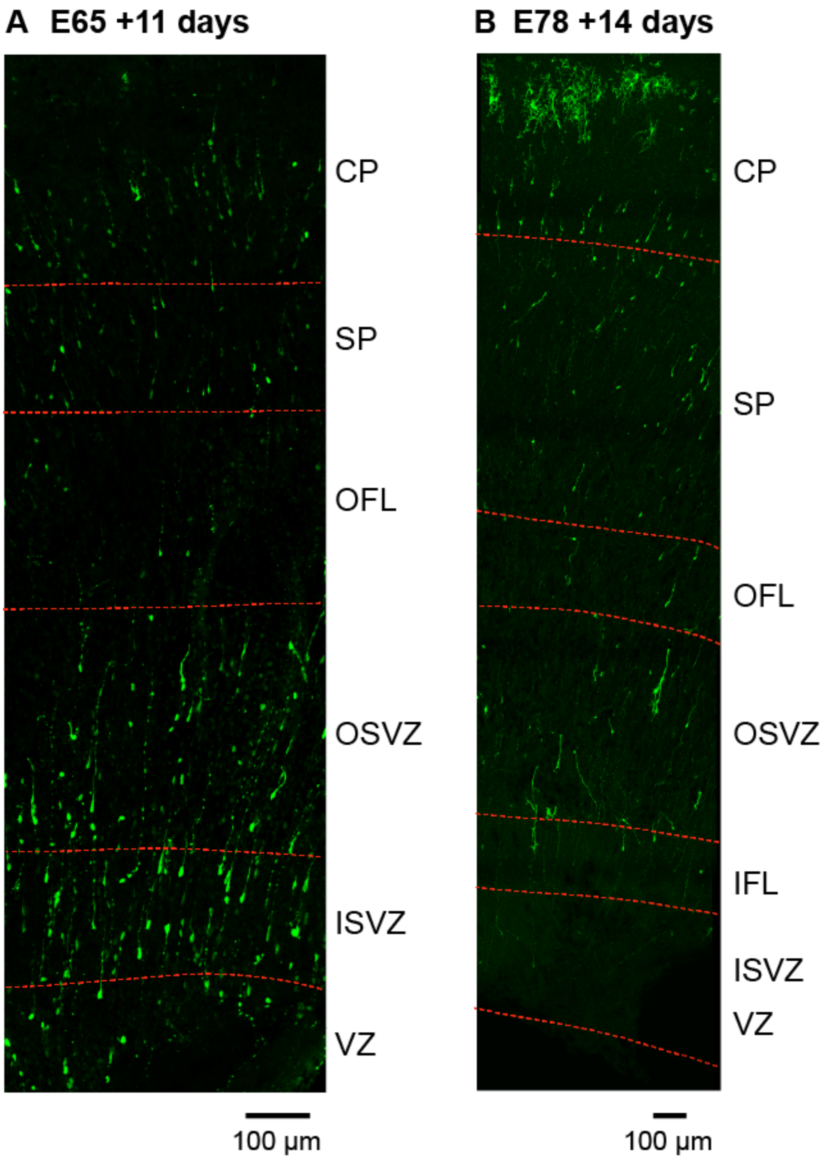

Figure S1. (**A**) Transect of a A17 parasagittal organotypic slice 11 days after EGFP retroviral infection of cycling precursors at E65. A fraction of EGFP+ neurons have migrated and reached the CP. (**B**) Transect of a A17 parasagittal organotypic slice 14 days after retroviral infection at E78. EGFP+ neurons have reached the CP. VZ: ventricular zone, ISVZ: inner subventricular zone, IFL: inner fiber layer, OSVZ: outer subventricular zone, OFL: outer fiber layer, SP: subplate, CP: cortical plate.

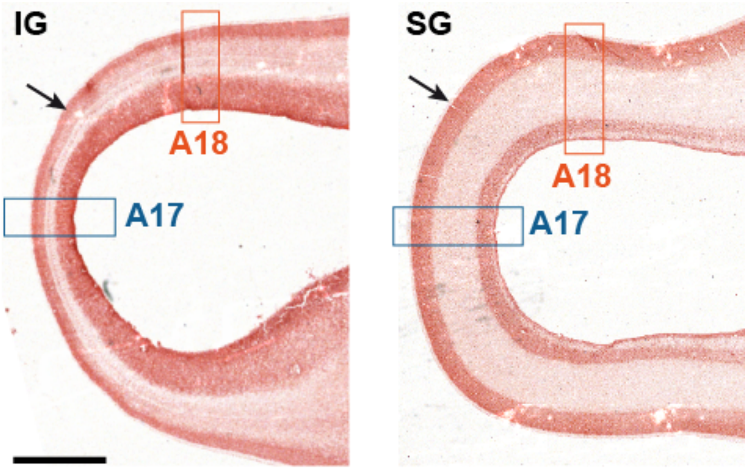

Figure S2. Parasagittal transects of the embryonic occipital lobe. Blue and orange frames correspond to A17 and A18 regions respectively where observations were implemented, in non-folded regions. Neutral red staining. IG (E65), SG (E78). Scale bar: 2 mm.

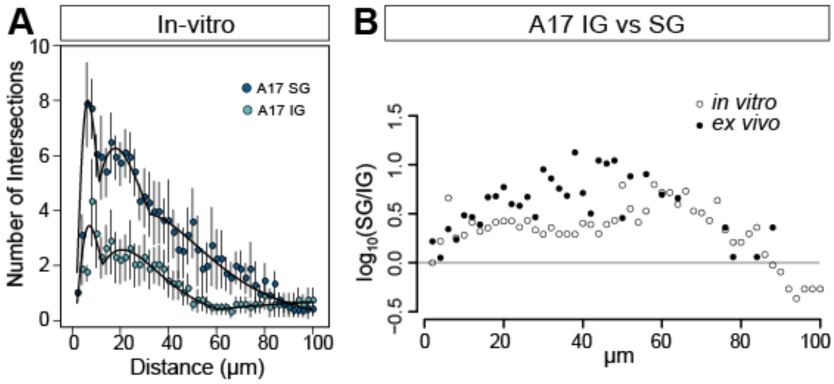

Figure S3. (**A**) Sholl analysis comparison between A17 IG (light blue) and A17 SG (dark blue) in dissociated cells. (**B**) Graph showing the Log ratio of A17 IG to SG neuronal densities (intersections/ sampling volume) as a function of distance for *in vitro* and *ex vivo* conditions. The relationship between IG and SG is stable over distance under the two experimental conditions. (A) Average value +/- sem.

2) Supplemental movies

Supplemental Movie 1: 2-photon imaging of SG migration in A17 area.
The cortical slices were infected with an EGFP retrovirus and the postmitotic neuron migration was imaged in real time, 4 days after infection. The acquisitions were done every 1.5h. The movie runs over a 142.5 hours period. Related to Figure 2.

Supplemental Movie 2: A17 SG neuron migration behavior.
Example of A17 neuronal migration on slices using 2-photon imaging. An infected EGFP expressing neuron is imaged during its migration from the OSVZ to the SP over 49.5 hours. The acquisitions were done every 1.5h. The red arrow points the cell body and turns blue when the neuron is pausing (pausing time: 9 hours). Related to Figure 2.

3) Key resources table

REAGENT or RESOURCE	SOURCE	IDENTIFIER
**Antibodies**		
Chicken anti-EGFP	Invitrogen	A10262
Rabbit anti-Ki67	Neomarker	RM9106S1
Mouse anti-Vimentin	Sigma	V6630
Mouse anti-NeuN	Millipore	MAB377
**Biological Samples**		
E60 to E82 primate brain slices and tissue	Laboratory	N/A
**Chemicals, Peptides, and Recombinant Proteins**		
DAPI	Molecular Probes	D1306
**Experimental Models: Cell Lines**		
**Experimental Models: Organisms/Strains**		
Macaque cynomolgus (macaca fascicularis)	Noveprim Europe	Camarney SL, Spain
**Recombinant DNA**		
pCMV-mCherry	Clontech	PT3974-5
pCMV-EGFP retrovirus	Betizeau et al., 2013	N/A
**Software and Algorithms**		
Image J 1.53c		Wayne Rasband, NIH, USA
MTrackJ (Image J)	NIH, USA	http://rsb.info.nih.gov/ij/
R OpenSource Statistical Software, glm (Generalized Linear Model)	Vienna, Austria	https://www.r-project.org
R MASS package	Venables and Ripley, 2002	http://www.stats.ox.ac.uk/pub/MASS4
R segmented package	Muggeo, 2008	https://cran.r-project.org/doc/Rnews/

Contact for Reagent and Resource Sharing

Reagents request must be addressed to Colette Dehay (colette.dehay@inserm.fr).

5) n values

Figure	n
1G	IG : 435 cells ; SG : 848 cells
2C/2D – A17 SG	2 brains, 2 hemispheres, 5 slices, 21 cells
2C – A17 IG	1 brain, 1 hemisphere, 3 slices, 8 cells
2D – A18 SG	2 brains, 2 hemispheres, 4 slices, 19 cells
2F	IG: 1 brain, 2 hemispheres, OFL: 81 cells, SP: 166 cells<br>SG: 1 brain, 1 hemisphere, OFL: 207 cells, SP: 242 cells

2G	IG: 1 brain, 2 hemispheres, OFL: 83 cells, SP: 158 cells<br>SG: 1 brain, 2 hemispheres, OFL: 141 cells, SP: 308 cells
2H – A17	IG: 1 brain, 2 hemispheres, 166 cells; SG: 1 brain, 1 hemisphere, 242 cells
2H – A18	IG: 1 brain, 2 hemispheres, 158 cells; SG: 1 brain, 2 hemispheres, 308 cells
3F	A17 : 2 brains, 2 hemispheres, 151 cells ; A18 : 2 brains, 3 hemispheres, 169 cells
3G	2 brains, 2 hemispheres, A17: 46 cells; A18: 44 cells
3H	2 brains, 2 hemispheres, A17: 44 cells; A18: 44 cells
3J	A17: 2 brain, 3 sections, 125 ROI; A18: 2 brain, 3 sections, 119 ROI
4E/4J	A17: 23 tracks; A18: 23 tracks
4F/4G	A17: 9 tracks; A18: 8 tracks
4I	A17: 10 tracks; A18: 13 tracks
4L	10 cells, 29 protrusions
4M	A17: 14 cells, 32 protrusions; A18: 10 cells, 23 protrusions
S3A	IG : 1 brain, 1 experiment, 7 cells ; SG : 2 brains, 3 experiments, 13 cells
